# Parental history of coronary artery disease among adults with hypothyroidism: Case controlled study

**DOI:** 10.1016/j.amsu.2020.10.034

**Published:** 2020-10-23

**Authors:** Hishyar Azo Najeeb, Bayar Ahmad Qasim, Ayad Ahmad Mohammed

**Affiliations:** a Department of Medical Chemistry, College of Medicine, University of Duhok, Kurdistan Region, Iraq; b Department of Medicine, College of Medicine, University of Duhok, Azadi Teaching Hospital, Duhok, Iraq; c Department of Surgery, College of Medicine, University of Duhok, Azadi Teaching Hospital, Duhok, Iraq

**Keywords:** Retinol binding Protein-4, Coronary artery diseases, Malondialdehyde, High-sensitivity CRP, Lipid peroxidation by-product

## Abstract

**Background:**

Thyroid dysfunction has a negative impact on coronary artery diseases (CAD) through several changes in its risk factors like dyslipidemia, glucose intolerance, and components of metabolic syndrome. Parental history of premature CAD may be an important risk factor for their offspring.

**Objective:**

To investigate whether overt and subclinical hypothyroidism and the risk of atherosclerosis are present in adults with parental history of CAD.

**Materials and methods:**

This case control study included 135 hypothyroid patients and 100 age-sex matched controls. Data were analyzed regarding CAD risk factors, hormonal and biochemical measures including retinol Binding Protein-4, fasting serum insulin, high-sensitivity C-reactive protein, lipid profile, fasting serum glucose, and serum malondialdehyde.

**Results:**

Parental history of CAD was significantly higher in overt hypothyroidism than subclinical group (P = 0.001). The level of RBP-4 in hypothyroid patients was significantly higher than euthyroid subjects (P = 0.03), and was higher in hypothyroid patients with positive parental history of CAD (p = 0.01). There were positive relationships between RBP-4 and related cardiovascular risk factors and with hypothyroidism, its sensitivity and specificity were 47.9% and 42.5% respectively. The positive predictive value was 60.8% and the negative predictive value was 30.4%. Hypothyroid patients with parental history of CAD had a risk of 3.7 times more than the euthyroid subjects.

**Conclusions:**

In hypothyroidism patients, parental history of CAD is a predictor of future coronary events and the related risk factors. RBP-4 is positively correlated with waist circumference, BMI, lipid profile, High-sensitivity CRP, MDA, fasting serum glucose, fasting serum insulin, HOMA indices and TSH.

## Highlights

• Thyroid dysfunction has a negative impact on coronary artery diseases.
• Parental history of premature CAD may be an important risk factor for their offspring.
• RBP-4 have been analyzed and shown to impair insulin sensitivity.

## 1 Introduction

The offspring of patients with premature coronary artery disease (CAD) may be at increased risk of atherosclerosis. Risk factors for atherosclerosis include age, male gender, family history of premature CAD, diabetes mellitus (DM), hypertension, smoking, high total and low density lipoprotein cholesterol, low high density lipoprotein cholesterol and obesity [1].

Several clinical studies have evaluated the natural history of atherosclerosis and shown higher serum concentration of lipoproteins, high sensitive C-reactive protein and higher blood pressure-risk factors for CAD in young adults with parental history of coronary disorder than those without such a background. The association between thyroid dysfunction and coronary heart disease has been well documented [2].

Overt hypothyroidism is usually presented as a stereotyped and early recognized syndrome with prominent features and clinical signs, while subclinical hypothyroidism can be asymptomatic [3].

Hypothyroidism (overt and subclinical) may change several risk factors such as dyslipidemia, hypertension, insulin resistance, glucose intolerance, obesity, inflammation and components of metabolic syndrome which may due to the regulatory effect of thyroid hormones on the role and action of some key enzymes of lipoproteins metabolism. However, not all patients with hypothyroidism have these conventional risk factors for CAD, suggesting that other factors may be involved. Parental history of premature CAD seems to be an important potential risk factor, and evidence suggests that positive family of CAD may increase cardiovascular risk [4,5].

Adipocytes secrete a variety of biologically active mediators (adipocytokines) which contribute to insulin resistance, type 2 DM and cardiovascular disease. Later on a new factor derived from fat cells, called retinol binding protein-4 (RBP-4) have been analyzed and shown to impair insulin sensitivity in the body and associated with various components of metabolic syndrome in diabetic subjects [[6], [7], [8]].

Prospective studies have shown that cardiovascular diseases aggregate in families, this is probably due in part to familial aggregation of important cardiovascular risk factors such as hypertension, obesity and high total serum cholesterol and low density lipoproteins (LDL) cholesterol. It has been demonstrated that both genetic and environmental factors contribute to the variability of risk factors and their familial aggregation [9,10].

Cardiovascular manifestations are a frequent finding in hypothyroidism state. Overt hypothyroidism is associated with increased risk for CAD, through an acceleration of atherosclerosis as indicated by hypertension, hyperlipidemia, increased LDL-cholesterol levels, and other components of metabolic syndrome. The prevalence rates for hypertension, hyperlipidemia, and undesirable lipoprotein profile, were also found in subclinical hypothyroidism [2,11,12].

Coronary artery atherosclerosis is twice as common in patients with hypothyroidism, the hypothesis of a causal relationship between hypothyroidism and atherosclerosis was first raised in 1883 by E. Theoder Kocher who noted that atherosclerosis commonly occurred after thyroid extirpation [13].

Subclinical hypothyroidism certainly influences cardiovascular risk; however, the relationship with atherosclerosis is still a matter of debate, this may result in acceleration of atherosclerosis and CAD, presumably because of associated hypercholesterolemia and hypertension, while the direct evidence of such effects of overt and subclinical hypothyroidism is lacking, however, in a study of 1149 postmenopausal women in Netherlands, those with subclinical hypothyroidism were more likely to have a history of myocardial infarction [14,15].

Atherosclerosis is a complex disease of the arterial walls, which may progress or regress depending on the capacity and severity of atherogenic risk factors. The classical risk factors for atherosclerosis in general population are age, male gender, family history of premature cardiovascular disease, diabetes mellitus, hypertension, smoking, high LDL-cholesterols, low HDL-cholesterol, and obesity. More recently, several novel risk factors such as insulin resistance (IR), oxidized LDL-cholesterols (oxLDL-ch), lipoprotein, homocysteine, hypertriglyceridemia, have been associated with increased risk of CAD [1,16,17].

There are six major component of the metabolic syndrome; abdominal obesity, atherogenic dyslipidemia, elevated blood pressure, insulin resistance ± glucose intolerance, a proinflammatory state, and aprothrobotic state or low fibrinolytic activity. At least three organizations have recommended clinical criteria for the diagnosis of the metabolic syndrome; the World Health Organization (WHO), the American Association of Clinical Endocrinologist (AACE) and the National Cholesterol Education Program (NCEP), and Adult Treatment Panel III (ATPIII). The clinical criteria proposed by ATPIII are abdominal obesity, elevated triglyceride, low HDL-cholesterol, high blood pressure, and high fasting glucose. When three of five of the characteristic are present, a diagnosis of metabolic syndrome can be made [18,19].

An increased adipose tissue mass is strongly associated with the pathogenesis of insulin resistance and type-2 diabetes mellitus. Adipose tissue may be viewed as an endocrine organ that secretes many types of adipokine (such as leptin, tumor necrosis factor alpha, interleukin-6, and adiponectin) that modulate the action of insulin in other tissues, moreover, RBP-4, a new fat-derived adipokine that specifically binds to retinol [20].

RBP-4 has been hypothesized to be a novel adipokine linking adiposity with systemic insulin resistance and potentially with adiposity-related disorders. RBP-4 positively correlated with GLUT-4 expression in adipose tissue, independently of any obesity variable. Circulating RBP-4 is similar in normal weight, overweight, and obese women. In insulin resistant state, the expression of GLUT-4 (the principal insulin-stimulated-glucose-transporter), is down regulated selectively in adipocytes and not in skeletal muscle; this result in impaired insulin stimulated glucose transport in adipocytes and this defect precedes glucose intolerance, however, the consequences of decreased GLUT-4 expression in adipocyte have been unclear, since adipose tissues contribute little to whole body glucose disposal [8,21,22].

### 1.1 Aim of the study

To investigate whether overt and subclinical hypothyroidism and the risk of atherosclerosis are present and are correlated in adults with a parental history of CAD**.**

## 2 Materials & methods

The study design was case-control which was done over a period of two years. Eligible subjects were enrolled by consecutive sampling procedure. Hypothyroid cases (135 patients) divided into 2 groups, the first group included 47 patients with overt hypothyroidism and with prominent signs and symptoms (TSH > 10.0 mIU/L and T4 < 66.0 nmol/L) and the second group included 88 patients with subclinical hypothyroidism (Asymptomatic) (TSH 4.5–10.0 mIU/L with normal or slightly lowered T4 levels), together with third group 100 euthyroid subjects designed as control group. From all hypothyroid cases and euthyroid subjects, the family history of coronary artery disease including premature myocardial infarction present in parents which is defined as family history of coronary heart disease in male first degree relative <55 years, coronary heart disease in female first degree relative <65 years [23].

Venous blood samples drawn in fasting state for biochemical laboratory analysis which included estimation of the total and high density lipoproteins, cholesterol, triglyceride, and glucose. These were measured by the Clinical Chemistry Auto-analyzer, Lisa.Xs (open system, automated, discrete, random access). Biocodes Hycel kit for total cholesterol BC4004/400, Biocodes Hycel kit for HDL-cholesterol R5BE50A, Biocodes Hycel kit for triglyceride R5B800A, Biocodes Hycel kit for glucose BC4003.

LDL-cholesterol was estimated by calculation using Friedewald formula. Serum RBP-4 was measured by ELISA Quantitation kit (GenWay Biotech, USA). Additional variables were estimated in our local laboratory (Department of Clinical Biochemistry) from serum aliquots series, stored at – 80 cº. Malondialdehyde (MDA) was estimated spectrophotometrically and Hs-CRP was assessed using commercial Chemiluminescent Immunometric Assay: LKCRP1, by IMMULITE 1000 Analyzers. Serum Insulin was assessed using a commercial kit (Elecsys insulin Kit: 12017547, Roche Diagnostics) by Roche/Hitachi Modular® analytics E 170 (Elecsys 2010) while insulin resistance was estimated by using Homeostatic model assessment for insulin resistance (HOMA-IR). Thyroid stimulating hormone (TSH) and serum thyroxine (T4) were assessed using Roche/Hitachi Modular® analytics E 170 (Elecsys 2010) (TSH kit: 11731459122, T4 kit: 12017709 122).

### 2.1 Statistical analyses

Categorical variables were described in frequency and percentage, while continuous variables were described in mean and standard deviation. P value of less than 0.05 was considered significant. The data were analyzed using the Statistical Package for Social Sciences (SPSS 25, IBM: USA).

### 2.2 Research registration

The research is registered according the World Medical Association's Declaration of Helsinki 2013 at the research registry at the 30th of August 2020, Research registry UIN: research registry **5969.**

The work of this article has been reported in line with the STROCSS criteria [24].

## 3 Results

One hundred and thirty-five hypothyroid patients together with 100 euthyroid subjects, were included in the study. They were age-sex matched. Baseline characteristics are presented in Table 1.

**Table 1:** Baseline characteristics of cases and controls.

Variables	Hypothyroid patients (n = 135)	Euthyroid Subjects (n = 100)	P.value
Age in years	32.6 ± 5.7	31.0 ± 7.1	0.07
Parental history of CAD Yes/No	45/90 *	12/88	0.001
RBP-4(ng/ml)	8.0 ± 4.8	5.7 ± 3.6	0.03
TSH(mIU/L)	18.3 ± 13.6	2.1 ± 1.0	0.001
T4(nmol/L)	76.3 ± 26.9	87.7 ± 16.3	0.001
Hs-CRP(mg/L)	7.2 ± 4.6	3.8 ± 2.7	0.001
Fasting serum insulin(μU/ml)	13.7 ± 9.8	8.5 ± 7.1	0.007
HOMA-IR	3.2 ± 2.0	1.7 ± 1.0	0.001
HOMA- β cell. %	52.5 ± 23.4	35.6 ± 14.0	0.04
Fasting serum glucose(mg/dl)	108.6 ± 26.2	82.4 ± 19.9	0.001
Total serum cholesterol(mg/dl)	227.5 ± 56.9	172.0 ± 21.3	0.001
Serum triglyceride(mg/dl)	161.7 ± 69.7	127.8 ± 55.2	0.001
HDL-cholesterol(mg/dl)	38.6 ± 8.9	47.9 ± 5.7	0.004
LDL-cholesterol(mg/dl)	150.0 ± 34.6	98.5 ± 0.5	0.001
LDL-Ch/HDL-Ch (ratio)	3.6 ± 1.0	2.6 ± 0.5	0.01
Serum MDA(nmol/L)	1.9 ± 0.5	1.0 ± 0.4	0.02
Values are Mean ± Standard Deviation unless indicated otherwise - *Chi-square test for difference between hypothyroid patients and euthyroid subjects. Abbreviations: HOMA-IR: Homeostatic Model Assessment for Insulin Resistance, MDA: Malondialdehyde.			

In the study population, the thyroid stimulating hormone (TSH) along with RBP-4 levels were significantly elevated in the hypothyroid patients compared with the euthyroid subjects, and they had high sensitivity C-reactive protein, lipid profile, malondialdehyde (MDA), fasting serum glucose, fasting serum insulin, and insulin indices levels than in euthyroid subjects. In addition, high density lipoprotein (HDL) was significantly lower in the first group when compared with the second one.

**Fig. 1:**
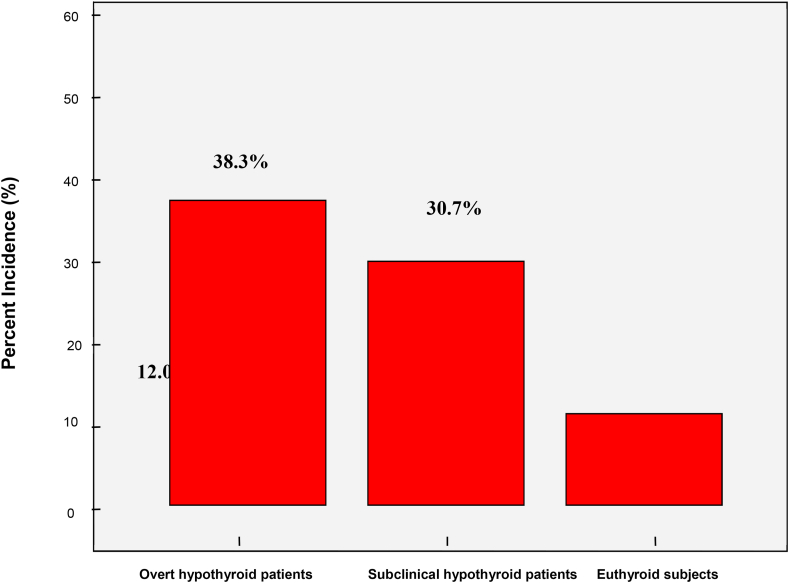
Distribution of positive parental history of coronary artery disease by thyroid status.

Frequency of parental history of coronary artery disease among overt hypothyroid, subclinical hypothyroid, and euthyroid groups is shown in Fig. 1.

**Fig. 2:**
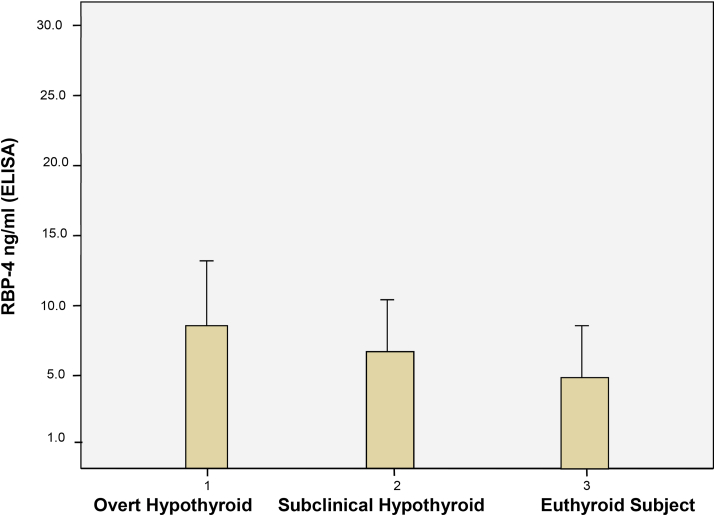
Retinol Binding Protein-4 levels by thyroid status.

Fig. 2 shows the comparison of retinol Binding Protein-4 (RBP-4) levels between patients with overt hypothyroidism, subclinical hypothyroidism and euthyroid subjects. Patients with overt and subclinical hypothyroidism had elevated RBP-4 (mean 8.9  ±  5.6 ng/ml, mean 7.1  ±  3.8 ng/ml respectively) that were statistically significant when compared with those of euthyroid patients (5.7  ±  3.6 ng/ml).

The distribution of the studied risk factors classified by thyroid status of the participants showed that 61.7% of overt hypothyroid patients and 44.3% of subclinical hypothyroid patients had central obesity compared to 32.0% of the euthyroid subjects (p < 0.001). Table 2.

**Table 2:** Distribution of related risk factors by thyroid status.

Risk factors	Overt hypothyroid patients (n = 47)	Subclinical hypothyroid Patients (n = 88)	Euthyroid subjects (n = 100)
Positive parental history of CAD	18 (38.3%)**	27 (30.7%)**	12 (12.0%)
Central obesity	29 (61.7%)*	39 (44.3%)*	32 (32.0%)
Obesity and/or overweight	37 (78.7%)*	65 (73.9%)*	58 (58.0%)
Hyperlipidemia	42 (89.4%)*	58 (65.9%)*	16 (16.0%)
Glucose intolerance	14 (29.6%)*	18 (20.5%)*	2 (2.0%)
Insulin resistance	10 (21.3%)**	13 (14.8%)**	1 (1.0%)
Hypertension	8 (17.0%)*	5 (5.7%)*	4 (4.0%)
Metabolic syndrome	13 (27.6%)**	23 (26.1%)**	4 (4.0%)
Based on X^2^ test, *p < 0.01, **p < 0.001			

Higher level of serum RBP-4 was seen in the patient group with positive parental history of CAD than in negative group (mean 9.9  ±  4.7 ng/L Vs 6.5  ±  4.5 ng/L, P = 0.01). As expected, total serum cholesterol, triglyceride, low density lipoprotein-cholesterol, and the atherogenic index (LDL-Ch/HDL-Ch ratio) were significantly higher than those of negative parental history of CAD. Regarding the lipid peroxidation by-product MDA and the specific marker of inflammation (high-sensitivity CRP); MDA and Hs-CRP levels of patients with positive parental history of CAD were higher than those of the negative group (p = 0.03, p = 0.01 respectively). There were no significant differences between the two groups regarding TSH, T4, fasting serum insulin, Homeostatic Model Assessment for Insulin Resistance (HOMA-IR), HOMA-% β-cell, HDL-cholesterol and fasting serum glucose. Table 3.

**Table 3:** Mean ± SD of the variables assessed in hypothyroid patients classified by parental history of CAD.

Variables	Patients with positive history (n = 45)	Patients with negative history (n = 90)	P value *
RBP-4 (ng/ml)	9.9 ± 4.7	6.5 ± 4.5	0.01
TSH(mIU/L)	18.3 ± 11.9	17.6 ± 9.2	0.5
T4 (nmol/L)	74.8 ± 25.7	76.7 ± 27.2	0.6
Hs-CRP (mg/L)	8.5 ± 4.0	6.6 ± 4.8	0.01
Fasting serum insulin (μU/ml)	15.4 ± 10.5	12.8 ± 9.4	0.2
HOMA-IR	3.4 ± 2.3	3.0 ± 2.4	0.4
HOMA- β cell. (%)	59.9 ± 23.1	48.6 ± 31.2	0.09
Fasting serum glucose (mg/dl)	101.2 ± 20.3	99.4 ± 24.5	0.6
Total serum cholesterol (mg/dl)	254.8 ± 52.9	213.8 ± 48.5	0.001
Serum triglyceride (mg/dl)	192.4 ± 61.6	146.4 ± 62.7	0.001
HDL-cholesterol (mg/dl)	42.7 ± 7.8	44.0 ± 9.4	0.4
LDL-cholesterol (mg/dl)	167.9 ± 63.6	141.2 ± 42.1	0.04
LDL-Ch/HDL-Ch (ratio)	4.0 ± 1.7	3.4 ± 1.4	0.02
Serum MDA (nmol/L)	2.1 ± 1.4	1.6 ± 0.6	0.03
* Based on *t*-test			

Table 4 shows that serum total cholesterol, triglyceride, LDL-cholesterol and LDL-cholesterol/HDL-cholesterol ratio were significantly higher in positive parental history group compared to negative family history group. Significantly higher mean values of serum RBP-4 concentration were demonstrated in subclinical hypothyroid patients with positive history compared to the group with negative history.

**Table 4:** Mean ± SD of the variables assessed in overt and subclinical hypothyroid patients classified by parental history of CAD.

Variables	Overt hypothyroid patients		Subclinical hypothyroid patients	
	Positive history (n = 18)	Negative history (n = 29)	Positive history (n = 27)	Negative history (n = 61)
R.B.P-4(ng/ml)	9.5 ± 6.1	8.5 ± 4.6	8.7 ± 2.6 *	6.1 ± 2.3
TSH(mIU/L)	43.7 ± 31.4	42.3 ± 29.1	6.0 ± 1.5	5.9 ± 1.4
T4(nmol/L)	54.7 ± 26.5	65.2 ± 36.4	81.0 ± 20.2	85.0 ± 19.7
Hs-CRP(mg/L)	9.0 ± 5.2	8.0 ± 6.7	7.7 ± 3.5	6.2 ± 3.4
Fasting serum insulin(μU/ml)	16.7 ± 11.8	15.0 ± 11.4	12.9 ± 8.1	11.0 ± 7.6
HOMA-IR	3.8 ± 2.3	3.6 ± 3.0	2.8 ± 1.6	2.5 ± 2.1
HOMA-%β-cell	66.8 ± 56.2	53.9 ± 47.6	50.8 ± 40.3	41.0 ± 30.2
Fasting serum glucose(mg/L)	111.3 ± 31.2	95.6 ± 12.7	101.0 ± 29.8	97.2 ± 18.7
Total Cholesterol(mg/dL)	326.2 ± 92.5*	246.3 ± 61.0	244.4 ± 67.5 *	2001 ± 35.0
Triglyceride(mg/dL)	309.4 ± 155.1*	190.3 ± 62.5	187.2 ± 61.6 *	131.4 ± 53.2
HDL-Ch.(mg/dL)	37.9 ± 7.2	44.7 ± 13.2	42.0 ± 7.9	43.5 ± 7.8
LDL-Ch.(mg/dL)	184.9 ± 75.6*	146.1 ± 39.4	155.3 ± 65.8 *	130.5 ± 30.8
LDL-Ch/HDLCh(Ratio)	4.8 ± 1.8*	3.7 ± 1.0	3.8 ± 1.7 *	3.1 ± 1.1
Serum MDA(nmol/L)	1.9 ± 0.8	1.6 ± 0.7	1.5 ± 0.3	1.2 ± 0.6
Based on *t-*test, *p < 0.05.				

As shown in Table 5, higher frequencies of central obesity, obesity and/or overweight, hyperlipidemia, glucose intolerance, insulin resistance, hypertension, and metabolic syndrome features were observed in hypothyroid patients with positive parental history of CAD, than those with negative parental history.

**Table 5:** Distribution of related risk factors in hypothyroid patients classified by parental history of CAD, n (%).

Risk factors	Patient with positive history (n = 45)	Patient with negative history (n = 90)	P.value*
Central obesity	33 (73.3%)	35 (38.9%)	0.001
Obesity and/or overweight	40 (88.9%)	62 (68.8%)	0.01
Hyperlipidemia	39 (86.7%)	61 (67.8%)	0.04
Glucose intolerance	13 (28.9%)	19 (21.1%)	0.4
Insulin resistance	11 (24.4%)	12 (13.3%)	0.1
Hypertension	8 (17.7%)	5 (5.6%)	0.052
Metabolic syndrome	23 (51.1%)	13 (14.4%)	0.001
* Based on X^2^ test			

Classifying hypothyroid patients by parental history of CAD revealed that the frequency of central obesity was 1.7-fold higher in positive parental history of overt and subclinical hypothyroid patients, than in negative group and increased 2.5-fold increase frequency of metabolic syndrome among them, Table 6.

**Table 6:** Distribution of related risk factors in overt and subclinical hypothyroid patients classified by parental history of CAD, n (%). The comparison between hypothyroid patients classified by gender is shown in Table 7.

Variables	Overt hypothyroid patients		Subclinical hypothyroid patients	
	Positive history (n = 18)	Negative history (n = 29)	Positive history (n = 27)	Negative history (n = 61)
Central obesity	15 (83.3%)*	14 (48.2%)	18 (66.7%)*	21 (34.4%)
Obesity and/or overweight	16 (88.9%)	21 (72.4%)	24 (88.8%)*	41 (67.2%)
Hyperlipidemia	17 (94.4%)	25 (86.2%)	22 (81.5%)*	36 (59.0%)
Glucose intolerance	6 (33.3%)	8 (27.5%)	7 (25.9%)	11 (18.0%)
Insulin resistance	7 (38.9%)*	3 (10.3%)	4 (14.8%)	9 (14.7%)
Hypertension	6 (33.3%)*	2 (6.9%)	2 (7.4%)	3 (4.9%)
Metabolic syndrome	8 (44.4%)*	5 (17.5%)	15 (55.6%)*	8 (13.1%)
Based on X^2^ test, *P < 0.05

**Table 7:** Mean ± SD of variables assessed in hypothyroid patients classified by gender.

Variables	Hypothyroid men (n = 21)	Hypothyroid women (114)	P.value*
RBP-4(ng/ml)	9.7 ± 4.9	7.5 ± 4.7	0.2
TSH(mIU/L)	27.5 ± 12.1	16.5 ± 11.7	0.06
T4(nmol/L)	70.4 ± 31.8	77.3 ± 25.9	0.3
Hs-CRP(mg/L)	8.3 ± 4.9	7.0 ± 4.6	0.2
Fasting serum insulin(μU/ml)	11.3 ± 6.7	12.1 ± 10.2	0.2
HOMA-IR	2.6 ± 1.5	3.2 ± 2.5	0.3
HOMA- β cell %	39.7 ± 25.7	54.3 ± 5.0	0.08
Fasting serum glucose (mg/dl)	96.7 ± 14.6	101.3 ± 25.5	0.2
Total serum cholesterol(mg/dl)	243.7 ± 54.4	224.5 ± 57.1	0.1
Serum triglyceride(mg/dl)	173.3 ± 70.5	159.9 ± 68.8	0.4
HDL-cholesterol(mg/dl)	44.9 ± 7.3	43.3 ± 9.2	0.3
LDL-cholesterol(mg/dl)	164.5 ± 52.2	147.4 ± 51.3	0.1
LDL-Ch/HDL-Ch (ratio)	3.7 ± 1.1	3.6 ± 1.6	0.7
Serum MDA(nmol/L)	1.8 ± 0.5	1.7 ± 1.0	0.9
* Based on *t*-test			

In estimation of the related risk factors of hypothyroidism patients with positive parental history of CAD, the whole female patients were divided into two groups, because the male sample size was small, 76.3% of female patients with positive parental history of CAD and 39.5% of those with negative history, had high central obesity and there was significant statistical difference between them (P < 0.001). The current frequency of obesity and/or overweight, hyperlipidemia, and metabolic syndrome features, showed the same tendency as that of high central obesity and was 15% higher in positive group (except for glucose intolerance and insulin resistance). Table 8.

**Table 8:** Distribution of related risk factors in hypothyroid females classified by parental history of CAD, n (%). Mean ± standard deviation of serum RBP-4 and related risk factor concentrations according to parental history in females are shown in Table 9.

Risk factors	Patient with positive history (n = 38)	Patient with negative history (n = 76)	P value*
Central obesity	29 (76.3%)	30 (39.5%)	0.001
Obesity and/or overweight	36 (94.7%)	52 (68.4%)	0.004
Hyperlipidemia	35 (92.1%)	51 (67.1%)	0.001
Glucose intolerance	12 (31.6%)	14 (18.4%)	0.1
Insulin resistance	7 (18.4%)	11 (14.5%)	0.7
Hypertension	6 (15.7%)	3 (3.9%)	0.001
Metabolic syndrome	18 (50.0%)	13 (22.4%)	0.001
* Based on X^2^ test

**Table 9:** Mean ± SD of the variables assessed in female hypothyroid patients classified by parental history of CAD.

Variables	Female patients with positive history (n = 38)	Female patients with Negative history (n = 76)	P.value *
R.B.P-4(ng/mL)	9.3 ± 4.1	6.6 ± 4.9	0.04
TSH(mIU/L)	16.9 ± 6.1	13.8 ± 8.7	0.07
T4(nmol/L)	76.7 ± 24.0	77.6 ± 6.9	0.8
Hs-CRP(mg/L)	8.0 ± 3.8	6.5 ± 4.9	0.04
F.serum insulin(μU/ml)	15.5 ± 10.8	13.4 ± 9.7	0.6
HOMA-IR	3.4 ± 2.4	3.1 ± 2.5	0.6
HOMA-%β-cell	60.9 ± 50.1	51.0 ± 42.7	0.3
F.serum glucose(mg/L)	101.9 ± 25.5	99.9 ± 25.8	0.6
T.cholesterol(mg/dL)	254.2 ± 33.5	209.7 ± 47.4	0.001
Triglyceride(mg/dL)	191.9 ± 45.1	143.4 ± 59.7	0.001
HDL-ch.(mg/dL)	42.0 ± 7.6	43.9 ± 9.8	0.2
LDL-ch.(mg/dL)	167.2 ± 65.5	137.5 ± 39.4	0.003
LDL-ch/HDLch(Ratio)	4.1 ± 1.8	3.3 ± 1.4	0.02
Serum MDA(nmol/L)	2.1 ± 1.5	1.6 ± 0.6	0.04
* Based on *t*-test			

**Fig. 3:**
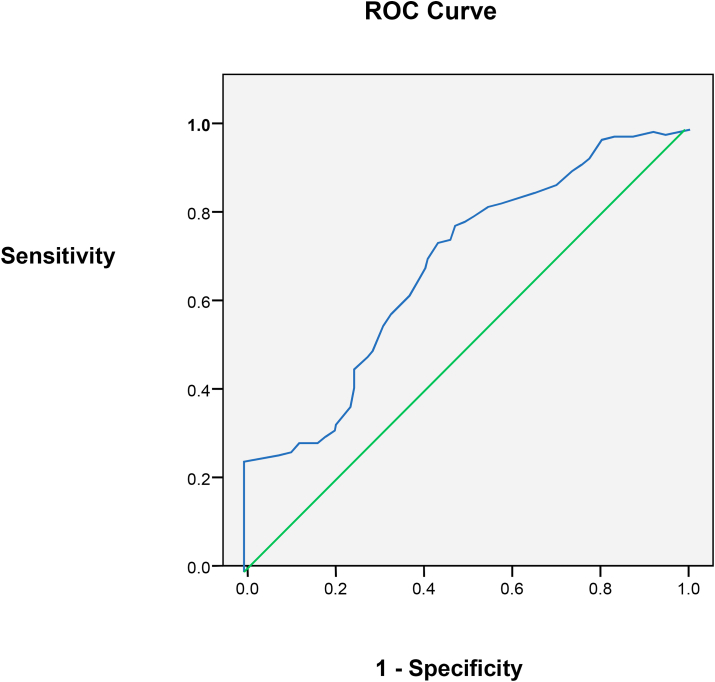
ROC curve showing the tradeoff between sensitivity (true positive) and false positive (1-specificity) of all available cutoff values for RBP-4 in predicting hypothyroidism.

To determine the performance characteristics (validity) of RBP-4 test in the diagnosis of hypothyroidism, ROC curve analysis was tested according to the trapezoidal rule. A sample of 24 patients with a diagnosis of overt hypothyroidism confirmed by TSH (>20 mIU/L) and T4 of (<60 nmol/L), and age range of (30–39years) were compared with a sample of 24 age-matched euthyroid subjects with TSH range of 0.4–4.0 mIU/L and T4 of 70–120 nmol/L (Fig. 3). The area under the ROC curve was estimated to be 0.675 with 95% CI of (0.523–0.827). At cutoff point of 7.0 ng/ml, sensitivity and specificity of RBP-4 were 47.9% and 42.5% respectively. The positive predictive value was 60.8% and the negative predictive value was 30.4% (Table 10). From these patients, 11 had positive parental history of CAD, with a mean RBP-4 of (9.6 ± 4.8) and 13 patients had negative parental history of CAD, with a mean RBP-4 (4.6 ± 3.2) the difference was statistically significant (P = 0.006).

**Table:** 

Area	Std. Error	Asymptotic Sig.	Asymptotic 95% confidence interval	
			Lower bound	Upper bound
.675	.078	.037	.523	.827

**Table 10:** Validity parameters for RBP-4 test in predicting hypothyroidism.

Positive if ≥ cutoff value	sensitivity	specificity	accuracy	PPV	NPV	LR
5.8	94.1	2.8	62.2	64.3	20.5	1.82
6.1	87.9	6.4	59.5	63.7	22.2	1.81
6.3	81.5	10.4	56.7	62.9	23.2	1.60
6.4	75.0	15.1	54.1	62.9	24.5	1.31
6.5	68.4	20.4	51.7	61.6	25.7	1.24
6.6	61.8	26.8	49.6	61.1	27.3	1.02
6.7	55.0	34.4	47.8	61.0	29.0	0.89
7.0	47.9	42.5	46.0	60.8	30.4	0.87
7.3	40.5	51.2	44.2	60.7	31.6	0.85
7.5	32.9	60.3	42.4	60.6	32.5	0.83
7.7	25.1	69.7	40.7	60.4	33.3	0.82
8.0	17.0	79.5	38.8	60.3	33.9	0.82
8.3	8.6	89.6	36.8	60.1	34.4	0.83

**Fig. 4:**
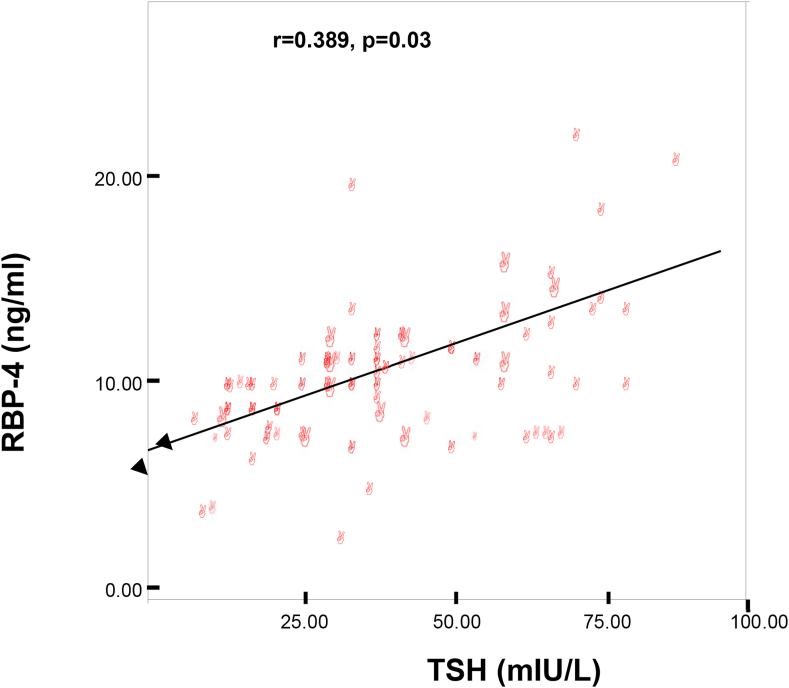
Correlation between RBP-4 and TSH in hypothyroid patients (n = 135).

All the related risk factors (except T4 and HDL-cholesterol) in hypothyroidism were significantly associated with RBP-4 when estimated adopting the spearman's coefficient: TSH r = 0.389, p = 0.003, fasting insulin r = 0.583, p = 0.001, insulin resistance r = 0.639, p = 0.001, fasting blood sugar r = 0.289, p = 0.028, total serum cholesterol r = 0.414, p = 0.001, serum triglyceride r = 0.589, p = 0.001, LDL-Ch r = 0.484 p = 0.001, LDL/HDL r = 0.521 p = 0.001, Hs-CRP r = 0.505 p = 0.001, MDA r = 0.411 p = 0.001. A similar relationship was observed in the overt and subclinical hypothyroid groups separately (Table 11) and (Fig. 4).

**Table 11:** Spearman's coefficient of RBP-4 with related variables in hypothyroid patients.

Groups	W.C	BMI	TSH	T4	F.insuln	Homa-IR	Homa-β%	FBS	T.Ch	TG	HDl	LDL	hsCRP	MDA
Total(n = 135) hypothyroidism	r 0.427 P 0.001	r 0.296 P 0.022	r 0.389 P 0.03	r 0.013 P o.924	r 0.583 P 0.001	r 0.639 P 0.001	r 0.371 P 0.005	r 0.289 P 0.028	r 0.414 P 0.001	r 0.589 P 0.001	r −0.1 P 0.19	r 0.484 P 0.001	r 0.505 P 0.001	r 0.411 P 0.001
Overt(n = 47) hypothyroidism	r 0.423 P 0.003	r 0.738 P 0.001	r 0.601 P 0.001	r 0.058 P 0.360	r 0.417 P 0.020	r 0.623 P 0.001	r 0.742 P 0.001	r 0.579 P 0.001	r 0.416 P 0.004	r 0.476 P 0.001	r −0.1 P 0.45	r 0.579 P 0.001	r 0.594 P 0.002	r 0.545 P 0.001
Subclinical(n = 88) hypothyroidism	r 0.387 P 0.01	r 0.358 P 0.02	r 0.427 P 0.001	r −0.08 P 0.508	r 0.484 P 0.01	r 0.500 P 0.001	r 0.364 P 0.01	r 0.251 P 0.044	r 0.264 P 0.038	r 0.399 P 0.002	r 0.12 P 0.35	r 0.364 P 0.018	r 0.504 P 0.001	r 0.324 P 0.012

Independent association between positive parental history of CAD and related risk factors were tested by multiple linear regression models using (Log) and RBP-4 as the dependent variable. The highest associations were found between RBP-4 and BMI, TSH, lipid profile, high sensitivity-CRP, lipid peroxidation by-product (MDA), waist circumferences, fasting insulin and HOMA indices (Except for systolic and diastolic blood pressure and T4 level), Table 12.

**Table 12:** Multiple linear regression analysis of related variables with RBP-4.

Independent variables	R	P.value
Waist circumference	0.547	0.005
Body mass index	0.795	0.001
Systolic Bp.	0.379	0.07
Diastolic	0.210	0.12
TSH	0.789	0.001
T4	0.200	0.33
Fasting serum insulin	0.457	0.04
HOMA-IR	0.465	0.03
HOMA-β%-cell function	0.453	0.04
Total s.cholesterol	0.876	0.001
Serum triglyceride	0.772	0.001
HDL-cholesterol	0.361	0.06
LDL-cholesterol	0.573	0.04
LDL-ch/HDL-ch	0.416	0.03
MDA	0.528	0.007
Hs-CRP	0.610	0.001
Fasting serum glucose	0.404	0.04

In multivariate analysis it was observed that the odds ratio were higher in hypothyroid patients when compared to euthyroid subjects for the following categories and as follow: for parental history of CAD (3.7), central obesity (2.1), obesity and/or overweight (2.2), hyperlipidemia (15.0), glucose intolerance (15.2), insulin resistance (20.3), metabolic syndrome (8.7) and hypertension (2.6). Table 13.

**Table 13:** Multivariate analysis of factors presumed to present a risk for coronary artery disease in hypothyroid patients compared to euthyroid subjects.

Variables	Hypothyroidism n (135)	Euthyroidism n (100)	X^2^	OR (95%CI)
Positive parental history of CAD	45 (33.3%)*	12 (12.0%)	13.0	3.7 (1.82–7.39)
Central obesity	68 (58.4%)*	32 (32%)	7.2	2.1 (1.26–3.7)
Obesity and/or overweight	102 (75.6%)*	58 (58.0%)	7.3	2.2 (1.28–3.91)
Hyperlipidemia	100 (74.0%)*	16 (16.0%)	75.2	15.0 (7.76–28.99)
Glucose intolerance	32 (23.7%)*	2 (2.0%)	20.1	15.2 (3.55–65.23)
Insulin resistance	23 (17.0%)*	1 (1.0%)	14.4	20.3 (12.7–153.31)
Hypertension	13 (10.6%)	4 (4.0%)	1.9	2.6 (0.81–8.09)
Metabolic syndrome	36 (26.7%)*	4 (4.0%)	19.3	8.7 (2.99–25.46)
*p < 0.05				

Regarding subclinical hypothyroidism, the odds ratio for parental history of CAD was 3.3. The odds ratio for other risk factors analyzed were 2.1 for obesity and/or overweight, 10.2 for hyperlipidemia, 12.6 for glucose intolerance, 17.2 for insulin resistance, 1.5 for hypertension and 8.5 for metabolic syndrome. Table 14.

**Table 14:** Multivariate analyses of factors presumed to present a risk for coronary artery disease between patients with subclinical hypothyroidism and euthyroid subjects.

Variables	Subclinical hypothyroidism n (88)	Euthyroidism n (100)	X^2^	OR (95%CI)
Positive parental history of CAD	27 (30.7%)*	12 (12.0%)	8.8	3.3 (1.53–6.90)
Central obesity	39 (44.3%)	32 (32%)	2.5	1.7 (0.93–3.06)
Obesity and/or overweight	65 (73.9%)*	58 (58.0%)	4.5	2.1 (1.10–3.80)
Hyperlipidemia	58 (65.9%)*	16 (16.0%)	46.8	10.2 (5.08–20.30)
Glucose intolerance	18 (20.5%)*	2 (2.0%)	14.9	12.6 (2.83–56.06)
Insulin resistance	13 (14.8%)*	1 (1.0%)	10.9	17.2 (2.20–134.1)
Hypertension	5 (5.7%)	4 (4.0%)	0.04	1.5 (0.38–5.56)
Metabolic syndrome	23 (26.1%)*	4 (4.0%)	16.9	8.5 (2.81–25.70)
*p < 0.05				

## 4 Discussion

In this study of hypothyroid patients, parental history of CAD was associated with lipid peroxidation, dyslipidemia, metabolic syndrome, hypertension, central obesity and obesity and/or overweight. The association was stronger when the overt hypothyroid patients were compared with subclinical hypothyroid. There was a significant difference in the serum levels of RBP-4 of the patients with parental history of present. Variability of risk factors were significantly higher for positive parental history than for negative parental history patients. These findings are consistent with 2 prospective –cohort studies that have examined association between the CAD and the thyroid dysfunction. Cappola and Ladenson examined the association of overt hypothyroidism with accelerated atherosclerosis and CAD, those authors reported increased risk of accelerated atherosclerosis and premature CAD in some patients [25].

Canturk et al. examined this correlation in subclinical hypothyroidism, a strong association between subclinical hypothyroidism and atherosclerotic cardiac disease, independent of the traditional risk factors has been reported. Recent studies have also suggested that thyroid diseases are associated with cardiovascular and peripheral vascular disease. Furthermore, data suggest the addition of family history information to risk prediction, based on traditional cardiovascular risk factors, however, little is known about the underlying parental history of CAD and the relation with coronary heart disease to thyroid dysfunction in young adults. The importance of parental history of CAD and related risk factors in hypothyroid patients are now discussed in turn [26].

Several clinical studies have shown positive correlation between adipokine RBP-4 levels and insulin resistance, BMI, and dyslipidemia. These studies suggest that an elevated RBP-4 levels may be associated with the cardiovascular risk factors that accompany insulin resistance in metabolic syndrome. On the other hand, hypothyroidism has been generally considered as cardiovascular risk factor in majority of studies, mainly because of its association with elevated serum total and low-density lipoprotein cholesterol. Hypercholesterolemia probably results from reduced catabolism of lipoproteins, a phenomenon that may be explained by a decreased expression of lipoprotein receptors. The present study ascertained that hypothyroid patients showed significantly higher RBP-4 concentrations by about 2.3 ng/ml [7,27,28].

It is also ascertained that hypothyroid patients with positive parental history of CAD showed significantly higher RBP-4 concentrations by about 3.4 ng/ml. The pathogenesis of elevated RBP-4 in hypothyroidism is generally attributed to the role of adipose tissue as an endocrine organ capable of secreting a number of adipose-tissue-specific hormones that are involved in the regulation of insulin action. RBP-4 has been suggested to be a novel adipokine linking obesity with systemic insulin resistance and potentially with adiposity-related disorders [29].

The association of hypothyroidism (overt and subclinical) with increased RBP-4 levels has also been reported. The finding of the present study is in line with that of who observed elevated RBP-4 in patients with overt hypothyroidism, but without coronary heart disease. The data of the present study showed that higher RBP-4 concentrations were associated with higher total cholesterol levels. Total cholesterol levels showed a trend toward higher values in the group with highest RBP-4 concentrations. Furthermore, other markers of glucose and lipid metabolism, including BMI, fasting glucose, fasting insulin, HOMA-IR, triglycerides, MDA and high sensitivity-CRP do correlate with RBP-4 serum concentrations in both overt and subclinical hypothyroid patients studied. Higher concentrations of RBP-4 were also observed in patients with positive parental history of CAD as well as hypercholesterolemia. In accordance with the present data, a positive association between RBP-4 and hypercholesterolemia is found in several studies [30,31].

In contrast, serum RBP-4 concentrations do not correlate with cholesterol, but with triglycerides in another study [8].

Overt hypothyroidism may result in accelerated atherosclerosis and CAD presumably because of the associated hypercholesterolemia. Thus, this association was completely independent of other factors, which are known to potentially stimulate the adipose tissue functions, notably from the observed increase of RBP-4. Structural and functional changes are present at an early age in the arteries of individuals with parental history of premature myocardial infarction. Part of this increased risk is due to clustering of cardiovascular risk factors, but in siblings, this elevated risk still persists after correction. The NCEP III guidelines provide such risk stratification based on many of these factors, such as smoking, diabetes mellitus, hypertension, obesity and lipids (total and low-density lipoprotein cholesterol, high-density lipoprotein cholesterol and triglycerides). In the present study the frequency of hyperlipidemia, obesity, hypertension, metabolic syndrome and elevated RBP-4 level was higher in hypothyroid patients with positive parental history of CAD than those with negative history, therefore increased RBP-4 in hypothyroid patients with positive parental history of CAD may be the cause of increased risk factors [32,33].

The question remains whether RBP-4 in hypothyroid patients could predict coronary risk. Data of the present study showed that at a cutoff point of 7.0 ng/ml, the positive predictive value was 46.0% (i.e. had positive parental history of CAD). The evidence of association of RBP-4 with coronary risk in addition to other risk factors of patients with positive parental history of CAD remains to be elucidated by epidemiological study.

The present study examined whether parental history of CAD is associated with cardiovascular risk factors in hypothyroidism. Despite low prevalence of positive family history of CAD (about 38.3% of overt hypothyroidism and 30.7% of subclinical hypothyroidism), an influence on any included cardiovascular risk factor in either overt or subclinical hypothyroid patients was found. Important association was identified among hypercholesterolemia, central obesity, hypertension insulin resistance, and metabolic syndrome in patients with overt hypothyroidism and in some cases with subclinical hypothyroidism. An original study showed that thyroid dysfunction and metabolic abnormalities including dyslipidemia, hypertension, insulin resistance, rising of inflammatory markers and modulation of other atherosclerotic factors provide partial explanation for how thyroid failure predisposes patients to cardiovascular disease. Surprisingly, no such association was considered in hypothyroid patients with parental history of CAD and this finding cannot be explained by unique influence of thyroid hormones [34].

Overt hypothyroidism is a well-known cause of dyslipidemia especially hypercholesterolemia and increases in low density lipoprotein cholesterol. Conversely, 4–14% of hypercholesterolemia patients have been reported to have thyroid dysfunction. Reports revealed that the percentage of patients with subclinical hypothyroidism having hyperlipidemia is 26.9%. In the present study the prevalence of hyperlipidemia in overt hypothyroidism was estimated to be 89.4% and in subclinical hypothyroidism was 65.9% in comparison to euthyroid group 16.0%. These high prevalence rates may indicate the presence of other factors in our population that accelerate the raising of total cholesterol and triglycerides additional to thyroid dysfunction [35,36].

The association between parental history of coronary heart disease and its associated risk factors, including measures of obesity, among offspring, has been documented in many studies of pediatric and young adult populations. Obesity is considered as a classical risk factor for cardiovascular disease in hypothyroid patients, which can predispose the inflammation and accelerate the atherosclerosis. The present study demonstrated an association between parental history of CAD and offspring BMI. Those who reported a positive history of CAD in either parents had a higher BMI and greater risk of being obese than those who reported no parental history. These findings confirm the reports of higher BMI and an increased prevalence of obesity among young adults with parental history of coronary artery disease [37].

To the best of our knowledge, previous studies among the young were based solely on the apparently healthy populations. The results can be explained by the fact that obesity, as a risk factor for CAD, is more prevalent among parents with a history of the disease, so their offspring adults become obese as well, and is more obvious in patients with thyroid dysfunction. The strong association between parental history of CAD and obesity should direct further studies towards the search for common genetic determinants of obesity and CAD, employing modern molecular epidemiological techniques, especially for subclinical hypothyroidism.

The metabolic syndrome and thyroid dysfunction are commonly associated with type 2 diabetes mellitus and cardiovascular disease. The results of the present study revealed a varied effect of thyroid status on the prevalence of metabolic syndrome. The observed changes were statistically significant within and between the overt hypothyroid, subclinical hypothyroid and euthyroid groups having parental history of CAD and the group not having history. The overall frequency of metabolic syndrome in hypothyroid group having parental history of CAD was 51.1% and the group not having history was 14.4%. In accordance with the present data metabolic syndrome and hypothyroidism are independent risk factor for cardiovascular disease. It is possible that patients suffering from both diseases may have a compounded risk [34,38,39].

Previous studies have indicated that insulin resistance and glucose intolerance contribute to increased cardiovascular atherosclerosis. The prevalence of glucose intolerance in thyroid disease has been estimated at 10.8% with the majority of cases occurring in overt hypothyroidism about (30%) and subclinical hypothyroidism (50%). In the present study, the frequency of glucose intolerance in hypothyroid patients was 24.0%, a similar increase in the prevalence of insulin resistance in hypothyroid patients was observed (17.0%). When compared between the groups, and there were high prevalence rates in the hypothyroid group with parental history of CAD; although the difference was not significant. Hyperinsulinemia is a good marker for insulin resistance which is frequently associated with other risk factors in hypothyroidism [40,41].

The association of hypothyroidism (both overt and subclinical) with increased C-reactive protein levels has also been reported. In contrast, other report did not show such association. In this study the serum levels of C-reactive protein were highly elevated in overt hypothyroid patients, particularly in those with parental history of CAD.

Positive parental history of CAD in offspring is clinically important aggregate marker of both heritable risk factors and as yet unmeasured genetic risk factors. There is substantial evidence linking family history of CAD with elevated lipid, high body weight, hypertension and elevated serum glucose [42,43].

The main limitation of our work is that large population based studies may better reflect the actual clinical status, and some other probable factors such as genetic, nutritional, and some life style habits may affects the coronary heart disease. Longer follow up is required to establish this possible correlation.

Similarly, the present study shows that these risk factors appear to be particularly more common among those hypothyroid patients with positive family history of CAD. Therefore, comprehensive assessment of such risk factors in those with positive parental history of CAD and those with negative history is crucial in present study. The study concludes that a larger study will consolidate the observations made in present study.

## Funding

The author was only financial supporter of the study.

## Conflicts of interest

There is no conflict of interest to be declared.

## Provenance and peer review

Not commissioned, externally peer reviewed.
